# Inflammatory monocytes and microglia play independent roles in inflammatory ictogenesis

**DOI:** 10.1186/s12974-022-02394-1

**Published:** 2022-01-29

**Authors:** Charles L. Howe, Reghann G. LaFrance-Corey, Brittany L. Overlee, Renee K. Johnson, Benjamin D. S. Clarkson, Emma N. Goddery

**Affiliations:** 1 Department of Neurology, Mayo Clinic, Rochester, MN 55905 USA; 2 Division of Experimental Neurology, Mayo Clinic, Rochester, MN 55905 USA; 3 Translational Neuroimmunology Lab, Mayo Clinic, Guggenheim 1542C, 200 First St SW, Rochester, MN 55905 USA; 4 Center for MS and Autoimmune Neurology, Mayo Clinic, Rochester, MN 55905 USA; 5 Mayo Clinic Graduate School of Biomedical Sciences, Mayo Clinic, Rochester, MN 55905 USA; 6 Present Address: Moderna, Cambridge, MA 02139 USA

**Keywords:** Theiler’s murine encephalomyelitis virus, Inflammatory monocyte, Microglia, Viral encephalitis, Hippocampus, Seizure, Epilepsy, Neuroinflammation

## Abstract

**Background:**

The pathogenic contribution of neuroinflammation to ictogenesis and epilepsy may provide a therapeutic target for reduction of seizure burden in patients that are currently underserved by traditional anti-seizure medications. The Theiler's murine encephalomyelitis virus (TMEV) model has provided important insights into the role of inflammation in ictogenesis, but questions remain regarding the relative contribution of microglia and inflammatory monocytes in this model.

**Methods:**

Female C57BL/6 mice were inoculated by intracranial injection of 2 × 10^5^, 5 × 10^4^, 1.25 × 10^4^, or 3.125 × 10^3^ plaque-forming units (PFU) of the Daniel’s strain of TMEV at 4–6 weeks of age. Infiltration of inflammatory monocytes, microglial activation, and cytokine production were measured at 24 h post-infection (hpi). Viral load, hippocampal injury, cognitive performance, and seizure burden were assessed at several timepoints.

**Results:**

The intensity of inflammatory infiltration and the extent of hippocampal injury induced during TMEV encephalitis scaled with the amount of infectious virus in the initial inoculum. Cognitive performance was preserved in mice inoculated with 1.25 × 10^4^ PFU TMEV relative to 2 × 10^5^ PFU TMEV, but peak viral load at 72 hpi was equivalent between the inocula. CCL2 production in the brain was attenuated by 90% and TNFα and IL6 production was absent in mice inoculated with 1.25 × 10^4^ PFU TMEV. Acute infiltration of inflammatory monocytes was attenuated by more than 80% in mice inoculated with 1.25 × 10^4^ PFU TMEV relative to 2 × 10^5^ PFU TMEV but microglial activation was equivalent between groups. Seizure burden was attenuated and the threshold to kainic acid-induced seizures was higher in mice inoculated with 1.25 × 10^4^ PFU TMEV but low-level behavioral seizures persisted and the EEG exhibited reduced but detectable abnormalities.

**Conclusions:**

The size of the inflammatory monocyte response induced by TMEV scales with the amount of infectious virus in the initial inoculum, despite the development of equivalent peak infectious viral load. In contrast, the microglial response does not scale with the inoculum, as microglial hyper-ramification and increased Iba-1 expression were evident in mice inoculated with either 1.25 × 10^4^ or 2 × 10^5^ PFU TMEV. Inoculation conditions that drive inflammatory monocyte infiltration resulted in robust behavioral seizures and EEG abnormalities, but the low inoculum condition, associated with only microglial activation, drove a more subtle seizure and EEG phenotype.

**Supplementary Information:**

The online version contains supplementary material available at 10.1186/s12974-022-02394-1.

## Background

Neuroinflammation is an emerging unifying mechanism underlying ictogenesis and the development of epilepsy in response to a diverse array of brain insults [1–3]. In general, neuroinflammatory responses involve both brain-resident effector populations, such as microglia [4] and astrocytes [5], and brain-infiltrating populations, such as inflammatory monocytes [6, 7] and neutrophils [8]. Despite recent work examining the relative contribution of these different populations to the initiation and propagation of seizures in several different animal models, the specific mechanistic roles for these cells are not yet fully understood [9–14]. Two dichotomous though not mutually exclusive populations are clearly involved in ictogenesis. Brain-resident microglia are rapidly activated in response to trauma, infection, and other brain insults, releasing cytokines, chemokines, danger signals, and other inflammatory mediators. In parallel, inflammatory monocytes rapidly infiltrate the brain in response to these same pathogenic drivers. Both populations employ similar effector mechanisms and may function antagonistically or synergistically, depending upon context and spatiotemporal interactions.

Viral encephalitis is an important cause of brain injury, ictogenesis, and temporal lobe epilepsy [15, 16]. While the vast majority of seizure and epilepsy disorders are not caused by virus infection of the CNS, the acutely amplified function of microglia and inflammatory monocytes observed during viral encephalitis is instructive regarding the role of these cells under non-infectious conditions. Indeed, a unique mouse model of viral encephalitis caused by intracranial inoculation of the Daniel’s strain of Theiler’s murine encephalomyelitis virus (TMEV) is associated with acute inflammation-induced seizures and the development of late spontaneous seizures [17–21]. We and others have shown in this model that brain-infiltrating inflammatory monocytes, responding to the chemoattractant CCL2 produced hyperacutely by neurons, drive profound damage to CA1 pyramidal neurons in the dorsal hippocampus and contribute to the generation of seizures via the production of inflammatory cytokines such as TNFα and IL-6 [22–27]. Recently, work led by Wolfgang Löscher has shown that microglia play a fundamental role in shaping the response to TMEV infection and provide an important protective or immunoregulatory function that may control other effector populations such as inflammatory monocytes [26, 28]. Of note, however, these investigators also reported that genetic deletion of CCR2, which prevents inflammatory monocyte infiltration and preserves the hippocampus during acute TMEV encephalitis, does not prevent the development of seizures. They interpret this to mean that infiltration of inflammatory monocytes is not required for ictogenesis in the TMEV model, a finding that is at odds with the pathogenic role we and others have established for this effector population.

To probe this disparity more closely we chose to ask the question, is the inflammatory monocyte response to viral inoculation necessary for seizures and can the ictogenic role of monocytes versus microglia be separated in the TMEV model by scaling the intensity of the monocytic infiltrate? To answer this, we inoculated mice with decreasing amounts of TMEV and assessed neuroinflammatory outcomes and pathological sequelae. Of note, TMEV is used in the field under various inoculation conditions, ranging from greater than 2 × 10^7^ PFU per mouse [26, 28] to 2 × 10^4^ PFU per mouse or lower [29], while our group and others routinely inoculate with 2 × 10^5^ PFU per mouse [22–25, 30–32]. In this study we found that the inflammatory monocyte response scaled with the amount of introduced virus while microglial activation did not. Moreover, we found that lower viral inoculum elicited a specific type of low-level behavioral seizures while a larger inoculum drove robust tonic–clonic type behavioral seizures. We propose that distinct viral inoculum-induced signals drive parallel activation of microglia and recruitment of inflammatory monocytes, leading to a complex interplay of these cells in ictogenesis and pathogenesis.

## Methods

### Virus

The Daniels (DA) strain of TMEV was used in this study. The 2nd and 3rd derivations of a 2nd generation viral stock originally derived from the 3rd generation of virus prepared by Moses Rodriguez at Mayo Clinic in 2007 were used in this work. The Rodriguez virus was derived from an original stock provided in the early 1980s by Lehrich and Arneson [33]. This virus was derived from the 5th passage of the original DA stock generated in 1952 by Joan Daniels [34]. Every rederived viral stock was validated on L2 cell monolayers. Quality control validation assays included replication in 4 week old B6 mice, induction of inflammatory monocyte and neutrophil infiltration at 24 hpi, and induction of VP2^+^ CD8 T cells at 7 dpi. Mice were inoculated by intracranial injection of 10 μL titered virus in RPMI growth media using a Hamilton syringe, as described [35]. The standard inoculum used by our group is 200,000 PFU per mouse. In this study, we employed fourfold serial dilutions of virus and 4 initial inocula: 200,000 PFU, 50,000 PFU, 12,500 PFU, and 3125 PFU per mouse.

### Mice

C57BL/6 and LysM:eGFP (both H-2^b^ haplotype) mice were maintained in-house, as described [24]. Mice were group housed in the Mayo Clinic research vivarium under conventional (non-barrier) conditions with ad libitum access to food and water. Females were used for all reported experiments. All animal experiments conformed to the National Institutes of Health guidelines and were approved by the Mayo Clinic Institutional Animal Care and Use Committee (Animal Welfare Assurance number A3291-01).

### Plaque assay for viral load

Mice were terminally anesthetized with isoflurane and then perfused with 30 mL PBS prior to removal of the brain and tissue weight determination. Following addition of 10 volumes (g:mL) cold DMEM, the tissue was dissociated by two 30-s bursts of a submerged rotor–stator homogenizer tip, on ice. The homogenate was clarified by centrifugation at 1000*g* for 20 min at 4 °C and stored in aliquots at − 80 °C. To establish viral titer, confluent monolayers of L2 fibroblasts (ATCC: CCL-149) grown in DMEM containing 10% FBS and 10 mM HEPES were washed once with serum-free DMEM and then adsorbed with tenfold serial dilutions of brain homogenate for 1 h at 37 °C, with gentle rocking every 10 min to distribute the media. At the conclusion of adsorption the cells were overlaid with 1 mL of 1% SeaPlaque agarose prepared in DMEM containing 2% FBS and 10 mM HEPES. After solidification at RT for 5–10 min the plates were incubated for 72 h at 37 °C, followed by fixation for 1 h in EtOH:HOAc:formaldehyde (6:2:1). The agarose plug was carefully extracted from the well, the fixed cell layer was washed with water, and the monolayer was stained with 1% crystal violet prepared in 20% EtOH for 5 min. After washing in water and air drying, the plaques were counted in triplicate at readable dilutions and the number was used to calculate PFU/g tissue.

### Brain-infiltrating leukocyte (BILs) isolation

BILs were isolated using two different methods. For both methods mice were terminally anesthetized with isoflurane and then perfused with 30 mL PBS prior to removal of the brain. In the first method, we followed our standard published procedure with slight modification [35]. The brain was homogenized in 5-mL ice-cold PBS by 10 strokes in a cold glass Tenbroeck homogenizer and brought to 10 mL final volume with PBS. A 30-mL round-bottom tube was prepared with 9 mL Percoll (GE Healthcare), 1 mL 10 × PBS, and 10 mL 1 × PBS at room temperature (RT). The 10 mL homogenate was added to this tube and mixed by inversion 3 times to yield a final 30% isotonic Percoll solution which was centrifuged at 7800*g* for 30 min at RT. The thick myelin-enriched debris layer at the top of the gradient was aspirated and the remaining homogenate solution was poured through a 40-μm filter into a 50-mL conical. The volume was brought to 50 mL with PBS and centrifuged at 800*g* for 5 min at RT. The pellet was resuspended in 1 mL PBS at RT, transferred to a glass round-bottom tube, underlaid with 1 mL 1.100 g/mL Percoll solution at RT, and centrifuged at 800*g* for 20 min at RT with no brake. The cells in the boundary layer were collected and washed in PBS prior to downstream processing.

In the second method, we used a modified enzymatic digestion protocol that is enriched in microglia and monocytes [36]. The brain was washed 3 times with 5 mL ice-cold low glucose DMEM containing 1% penicillin–streptomycin, then placed in a 60-mm Petri dish on ice containing 5 mL of the wash solution. The hemispheres were divided and the meninges removed with a fine forceps prior to chopping the brain into ~ 1 mm^2^ cubes with a scalpel blade. The pieces were digested in papain and DNase I diluted in the 5 mL wash solution for 30 min at 37 °C, followed by trituration with a P1000 pipette tip. The suspension was poured through a 100-μm filter into a 50-mL conical and pieces retained by the filter were broken up by grinding with the plunger of a 3-mL syringe while continuously adding wash solution until the final volume through the filter is 15 mL. The suspension was centrifuged at 500*g* for 5 min at 4 °C and the pellet was resuspended in 8 mL 30% Percoll prepared in DMEM, then underlaid with 70% Percoll prepared in HBSS. The gradient was centrifuged at 650*g* for 25 min at RT with slow acceleration and no brake. The debris layer at the top of the gradient was aspirated and approximately 3 mL of the cloudy 30:70 gradient interface was collected, diluted with 9 mL HBSS, and spun at 500 g for 5 min. The pellet was resuspended in DMEM for downstream processing.

### Flow cytometric cellular analyses

**Fig. 1:**
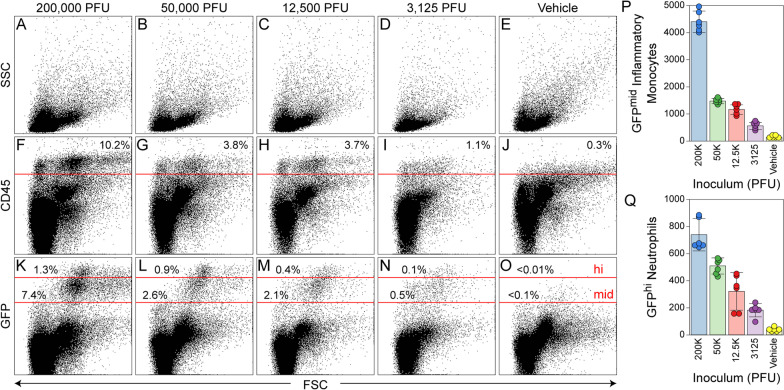
The intensity of the acute inflammatory infiltration response to TMEV is titrated by the concentration of the initial viral inoculum. BILs were isolated from LysM:eGFP reporter mice 24 h after intracranial inoculation with 200,000 PFU (**A**, **F**, **K**), 50,000 PFU (**B**, **G**, **L**), 12,500 PFU (**C**, **H**, **M**), or 3125 PFU (**D**, **I**, **N**) of TMEV or injection of vehicle (**E**, **J**, **O**). **A**–**E** Forward (FSC) and side (SSC) scatter profiles indicate the presence of more cells with larger FSC in the higher inoculum groups, though the overall number of cells isolated in the BILs preparations are similar. **F**–**J** The number of CD45^hi^ cells infiltrating the brain (above the horizontal line) scales with the initial inoculum, while the relative density of CD45^mid^ cells is similar across conditions. **K**–**O** The number of GFP^mid^ inflammatory monocytes (between the horizontal lines) also scales with the initial inoculum. **P** Quantitation of GFP^mid^ inflammatory monocytes and (**Q**) GFP^hi^ neutrophils. Each dot represents one animal. Bars show mean ± 95% confidence intervals. Percentages in **F**–**O** indicate CD45^hi^, GFP^hi^, and GFP^mid^ populations relative to total cells in the analysis

BILs were blocked in PBS containing 1% BSA and 25% (v:v) supernatant from the 2.4G2 hybridoma (anti-CD16/32; DMEM containing 5% horse serum and 5% FBS) for 30 min at 4 °C, then incubated for 30 min at 4 °C with fluorescently conjugated primary antibodies diluted 1:200 in block. After 3 washes cells were analyzed by flow cytometry using an Accuri C6 instrument (BD Biosciences). Data were post-processed in FlowJo 10.1 and re-presented using Photoshop CC 2017 and Illustrator CC 2017 (Adobe). The following primary antibodies were used, all from BD Biosciences: anti-CD45 = clone 30-F11 (#557235); anti-CD11b = clone M1/70 (#553312); anti-Ly6C/G = clone RB6-8C5 (#553129; Gr1); anti-Ly6G = clone 1A8 (#551467). Data in Fig. 1 are shown ungated. Data in Fig. 5 are derived from an initial CD45^+^ gate. Gates for inflammatory monocytes, neutrophils, and activated microglia are based on our published findings [24, 25] (Additional file 1: Fig. S1). Inflammatory monocytes are operationally defined as CD45^hi^CD11b^++^Gr1^+^1A8^−^; microglia are defined as CD45^mid^CD11b^+^Gr1^−^1A8^−^ [24, 25].

### Cytokine analyses

Mice were terminally anesthetized with isoflurane and then perfused via intracardiac puncture with 30 mL PBS. Hippocampi were microdissected, weighed, and disrupted in 500 μL PBS containing a protease inhibitor cocktail (10 μg/mL aprotinin, 1 μg/mL leupeptin, 1 mM phenylmethylsulfonyl fluoride, 1 mM NaF, 1 mM Na_3_VO_4_) using 10 strokes in a cold 2-mL glass Tenbroeck homogenizer. Following centrifugation at 14,000*g* for 10 min at 4 °C the clarified supernatants were stored at − 20 °C until use. Levels of CCL2, TNFα, and IL6 were measured using a mouse inflammatory cytokine cytometric bead array multiplex panel (#552364, BD Biosciences). Standard curves were included in all assays and samples were quantified using an Accuri C6 flow cytometer. Files were analyzed automatically using FCAP Array 3.0 (BD Biosciences) and manually using FlowJo 10.1, Microsoft Excel, and MATLAB R2017a (MathWorks).

### Histology, immunostaining, and microscopy

**Fig. 2:**
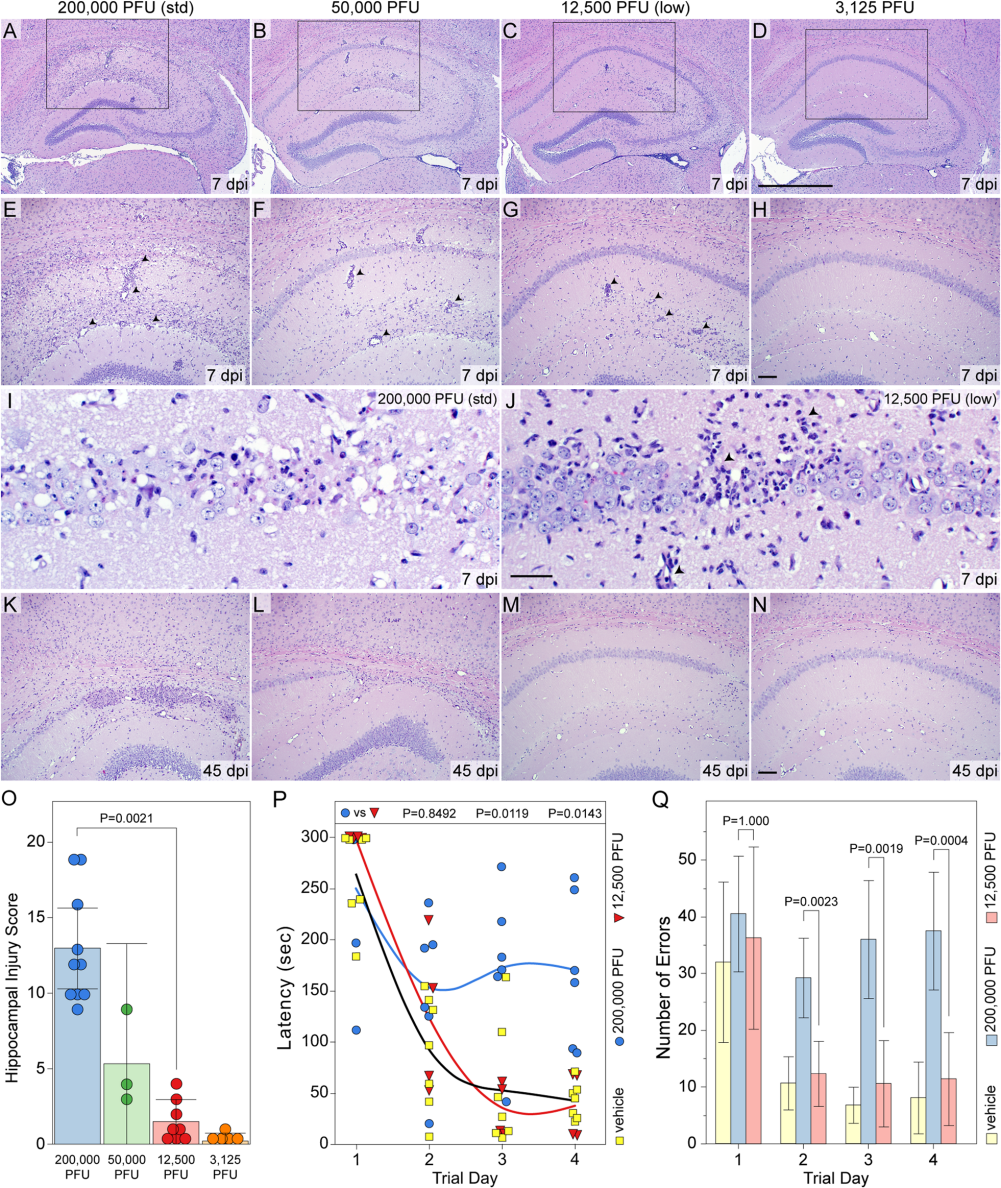
The extent of hippocampal injury and cognitive impairment is titrated by the concentration of the initial viral inoculum. Hippocampal pathology was assessed by H&E histology at 7 dpi (**A**-**J**) and 45 dpi (**K**-**N**) in mice inoculated with 200,000 PFU (**A**, **E**, **I**, **K**), 50,000 PFU (**B**, **F**, **L**), 12,500 PFU (**C**, **G**, **J**, **M**), or 3125 PFU (**D**, **H**, **N**) of TMEV. Extensive destruction of the CA1 pyramidal layer is observed at 7 dpi in mice inoculated with the highest concentration of TMEV (**A**, **E**, **I**, **O**). CA1 destruction is more variable but still readily apparent at 7 dpi in mice inoculated with 50,000 PFU of TMEV (**B**, **F**, **O**) and in some mice the extent of CA1 damage observed at 45 dpi (**L**) is as extensive as that observed in mice inoculated with 200,000 PFU of TMEV (**K**). Mice inoculated with 12,500 PFU of TMEV exhibit preservation of CA1 neurons at 7 dpi, with many mice exhibiting nearly complete sparing of CA1 (**C**, **G, O**) and only small, discrete areas of injury at high magnification (**J**). This preservation was verified at 45 dpi in the 12,500 PFU cohort (**M**). The absence of injury observed in this group occurs despite the presence of infiltrate apparent in the H&E stained sections (**C**, **G**, **J**). Finally, mice inoculated with 3125 PFU of TMEV exhibit essentially no evidence of CA1 injury at 7 dpi (**D**, **H**, **O**) or 45 dpi (**N**) and show only weak inflammatory infiltration. (**O**) Quantitation of hippocampal injury at 7 dpi reveals profound preservation in mice inoculated with 12,500 PFU of TMEV. *F*_(3,22)_ = 37.2861, *P* < 0.0001 by one-way ANOVA; Shapiro–Wilk *P* = 0.0020; Dunn's method pairwise analysis: 200,000 PFU vs 12,500 PFU: *P* = 0.0021; 200,000 PFU vs 3125 PFU: *P* = 0.0005; 12,500 PFU vs 3125: *P* = 1.000; *d*_s_ = 2.1; pooled results from 2 separate experiments. Mice were inoculated with 200,000 PFU or 12,500 PFU of TMEV and cognitive performance was assessed starting at 45 dpi using the Barnes maze (**P**–**Q**). **P** Latency to acquisition of the escape hole (s) was measured over 4 testing days in infected mice and compared to performance in uninfected mice inoculated with vehicle. Mice inoculated with 200,000 PFU do not learn to navigate to the escape while mice inoculated with 12,500 PFU perform as well as control mice. Latency: *F*_(11,56)_ = 16.6172, *P* < 0.0001 by two-way ANOVA (inoculum x day); Shapiro–Wilk *P* < 0.0001; Tukey HSD pairwise analysis: @ day 1: 200,000 PFU vs vehicle: *P* = 1.000; 12,500 PFU vs vehicle: *P* = 0.9999; 200,000 PFU vs 12,500 PFU: *P* = 0.9971; @ day 4: 200,000 PFU vs vehicle: *P* = 0.0109; 12,500 PFU vs vehicle: *P* = 1.000; 200,000 PFU vs 12,500 PFU: *P* = 0.0143; *d*_*s*_ = 0.55. Each symbol represents an individual animal. **Q** Analysis of error rate also indicates normal spatial memory acquisition in the mice infected with 12,500 PFU but not in mice inoculated with 200,000 PFU. Error rate: *F*_(11,56)_ = 18.9760, *P* < 0.0001 by two-way ANOVA (inoculum x day); Shapiro–Wilk *P* = 0.0011; Tukey HSD pairwise analysis: @ day 1: 200,000 PFU vs vehicle: *P* = 0.9190; 12,500 PFU vs vehicle: *P* = 0.9997; 200,000 PFU vs 12,500 PFU: *P* = 1.000; @ day 4: 200,000 PFU vs vehicle: *P* < 0.0001; 12,500 PFU vs vehicle: *P* = 0.9530; 200,000 PFU vs 12,500 PFU: *P* = 0.0004; *d*_*s*_ = 0.62. Scale bars: **D** = 1 mm (**A**–**D**); **H** = 100 μm (**E**–**H**); **J** = 100 μm (**I**, **J**); **N** = 100 μm (**K**–**N**). ** indicates *P* < 0.01 in panel (**O**). Arrowheads in **E**–**J** highlight representative examples of inflammatory infiltrate

Mice were terminally anesthetized with isoflurane and then perfused via intracardiac puncture with 30 mL PBS prior to perfusion with 50 mL of ice-cold, freshly prepared 4% paraformaldehyde (PFA). For paraffin processed tissue (Fig. 2), the brain was post-fixed for 24 h in PFA at 4 °C prior to macrosectioning the tissue with coronal cuts at the level of the optic chiasm and the infundibulum [22]. The 3 tissue blocks were embedded together in paraffin, cut at 5 μm, mounted on charged slides, rehydrated, and stained with hematoxylin and eosin, as described [25]. Loss of hippocampal neurons was quantified as previously described, with slight modification [31]. In brief, damage to the stratum pyramidale of each hemisphere was scored on coded slides by two blinded observers on a scale of 0–10, with 0 representing no injury and 10 representing complete loss of the pyramidal cell layer; if 20% of neurons were lost, a score of 2 was assigned. The scores from both hemispheres were summed and the average score between the two observers was taken as the score for the individual animal. The maximum injury score in any animal is 20.

For immunofluorescence analysis (Fig. 5), the brain was post-fixed in 4% PFA overnight at 4 °C, transferred to 30% sucrose, and infiltrated overnight. The entire brain was embedded in OCT (Tissue-Tek), frozen in cryostat molds using liquid nitrogen and isopentane, then stored at − 80 °C until use. Molds were equilibrated to the cryostat temperature (− 20 °C) for 20 min, then cut at 15 μm. Sections were adhered to gelatin-coated slides at RT, briefly warmed on a hot plate to increase adhesion and flattening, and stored at − 20 °C until use. Sections on slides were rehydrated for 10 min with PBS, then blocked in PBS containing 0.5% TX-100 and 2% normal donkey serum for 1 h at RT. Sections were incubated overnight at 4 °C in 0.17 μg/mL rabbit anti-Iba1 (#019-19741, Wako Chemical) primary antibody diluted in block. Following 3 washes in PBS the sections were incubated in 6.25 μg/mL Alexa Fluor 488-conjugated donkey anti-rabbit secondary (#711-545-152, Jackson ImmunoResearch Laboratories) diluted in block for 1 h at RT. After washing in PBS, sections were mounted with VECTASHIELD antifade mounting medium containing DAPI (Vector Laboratories). Images were collected using optical sectioning and structured illumination on a Zeiss Apotome.2 microscope equipped with an Axiocam 503 camera. The following parameters were employed: Alexa 488 channel: EX (493, 450–490), EM (517, 500–550), 700 ms exposure, 1.15 μm optical section, 7 slices); DAPI channel: EX (353, 335–383), EM (465, 420–470), 10 ms exposure, 1.03 μm optical section, 7 slices. Data were processed in Zen Blue (Zeiss) and post-processed in ImageJ (National Institutes of Health) and Photoshop CC 2017. Optical stacks were combined using a maximum projection algorithm. Individual data channels were histogram leveled before recombination using identical parameters for all experimental samples. No other manipulations were performed other than resizing and cropping for orientation.

### Behavioral testing

Spatial memory was assessed using a non-aversive Barnes-style platform maze [37] modified for mice [38]. A platform 92 cm in diameter with 20 evenly spaced holes distributed around the outer edge was placed on top of a second platform with one escape hole, permitting 360° rotation of the escape location relative to visual cues. Four spatial cues presented as white shapes on a black background were evenly distributed around the testing platform, just outside of the maze surface. Extraneous cues were suppressed by a black encircling curtain, thorough washing of the maze surface and escape chamber between animals, and by consistency in investigator behavior. The maze was brightly and evenly illuminated with an overhead ring light. Video was captured and analyzed using the EthoVision XT 10 system (Noldus). Following acclimatization to the testing room, mice were placed in the center of the maze and allowed 5 min to find the escape hole. Mice that failed to find the hole by 5 min were gently guided to the escape. After entering the escape chamber mice were given 1 min before removal and return to the home cage. Each animal experienced 3 trials, separated by 30 min, every day for 4 days, during which the escape location was static relative to the visual cues. The latency-to-escape (s) and the number of primary errors (investigation of a non-escape hole prior to discovery of the correct escape location) were determined for each trial [39]. On the 5th day the bottom platform was rotated 180° relative to the top platform and the visual cues, placing the escape hole in the quadrant opposite to the training location. Three probe trials, separated by 30 min, were performed to assess short-term reference memory retention. The mean probe trial latency was divided by the mean escape latency on the 4th regular trial day to generate a probe trial index. Mice that do not establish spatial reference memory exhibit an index of 1; larger indices are indicative of more time spent investigating the trained location of the escape hole prior to reverting to exploration to find the new escape location [40, 41].

### Bone marrow transplantation

Starting one week prior to bone marrow reconstitution and continuing for 2 weeks after, mice were provided acidified water (pH 2.5–3.0) and irradiated chow. Starting 5 days prior to tail vein injection mice were handled daily for 5 min and inserted into a restraint device without manipulation in order to reduce the stress response. On the day of reconstitution mice were irradiated with 400 rads of whole body radiation delivered over 10 min in a J.L. Shepherd & Associates Mark 1–25, 5000 Ci ^137^Cs irradiator. Three hours later mice received another 400 rads over 10 min. Donor mice were killed by isoflurane overdose and decapitation. Bone marrow was flushed from both femurs and tibias using ice-cold PBS, passed once through a 32-gauge needle, gravity filtered through a 30-µm strainer, washed in PBS, resuspended in ACK buffer (155 mM NH_4_Cl, 10 mM KHCO_3_, 0.01 mM EDTA in water) to lyse red blood cells, washed in RPMI containing 10% heat-inactivated FBS, and counted by trypan blue exclusion. Three hours after the second radiation dose recipient mice were briefly warmed under a heat lamp, immobilized, and the tail was swabbed with 70% EtOH. Mice received 10^7^ bone marrow cells via a 25-gauge needle inserted 2–4 mm into the tail vein lumen with the bevel facing upwards. Reconstitution proceeded for 4 weeks prior to experimental use.

### Seizure scoring

Behavioral seizures were quantified using a modified Racine scoring system [42], as we have previously published [43]. In brief, 5 cage mates were placed in an observation box for 1 h each day at 9 AM and 3 PM. An expert observer, blind to experimental condition, scored seizure manifestations using the following scale: 0 = normal behavior; 1 = freezing, orofacial twitching, repetitive orofacial grooming; 2 = repetitive head nodding or head twitching; 3 = unilateral forelimb clonus; 4 = rearing, bilateral forelimb clonus; 5 = falling, whole body tonic–clonic seizure activity. During the observation period, the maximal score during any 1-min window was recorded. Total daily seizure burden was calculated for each animal by summing the behavioral scores measured during the hour-long epochs. Total average daily seizure burden for each group was calculated by summing the seizure burdens for each animal on each day and dividing by the number of animals observed.

### Kainic acid-induced seizure threshold analysis

Kainic acid (Tocris #0222) was prepared at 5 mg/mL in basic water (1 drop 10 N NaOH per mL water). For each separate experiment (comparing uninfected and the 2 inoculation groups) adequate kainic acid stock was prepared for all animals [44]. On the day of testing, kainic acid was diluted to 1 mg/mL in PBS. Mice were weighed and marked on the tail with a black marker for identification. Individual syringes were prepared for each animal and each dose. Mice were placed in cohorts of 5 in an open top disposable cage with bedding under normal room lighting. At time zero mice received 5 mg/kg kainic acid by intraperitoneal injection (~ 30 s per mouse). A trained observer carefully observed the animals throughout the course of the experiment and rated behavioral seizures as described above. Subsequent kainic acid injections were delivered at 30, 60, 90, 120, and 150 min. All behavioral events were recorded for time of onset. Mice were removed from the experiment and euthanized after the initiation of the second independent episode of Racine 4/5 seizures and the time and number of doses were recorded. All mice fulfilled status epilepticus criteria after 6 or fewer doses of kainic acid.

### EEG

Mice were instrumented at 4–5 weeks of age following our established methods with minor modifications [43]. Briefly, animals were administered buprenorphine sustained release and then anesthetized with isoflurane in an induction chamber. After placement on the stereotactic rig and evidence of a surgical plane of anesthesia, an incision was made in the scalp to expose bregma and the midline suture. Screw electrodes wrapped with silver wire were screwed into burr holes placed at the following coordinates relative to bregma: channel 1: − 2.0 caudal, + 1.7 lateral; channel 2: − 2.0 caudal, − 1.7 lateral; reference: + 1.0 caudal, + 1.5 lateral; ground: ~ − 5.0 caudal, − 0.5 lateral. Head mounts (Pinnacle #8235-SM) were soldered to the wires connected to the screw electrodes. Dental cement was used to adhere the headmount and protect the recording electrodes. Following 1–2 weeks post-operative recovery mice were connected to a tethered Pinnacle Technologies pre-amplifier and data acquisition and conditioning system. Data were sampled at 1000 Hz either during 1-h epochs at the same time of day or continuously for several days. Following baseline collection, mice were inoculated with TMEV and recorded again at 72 hpi. Data were converted to European data format (.edf) and analyzed in Matlab using standard line length and power spectrum calculations.

### Experimental design and statistical analyses

**Fig. 3:**
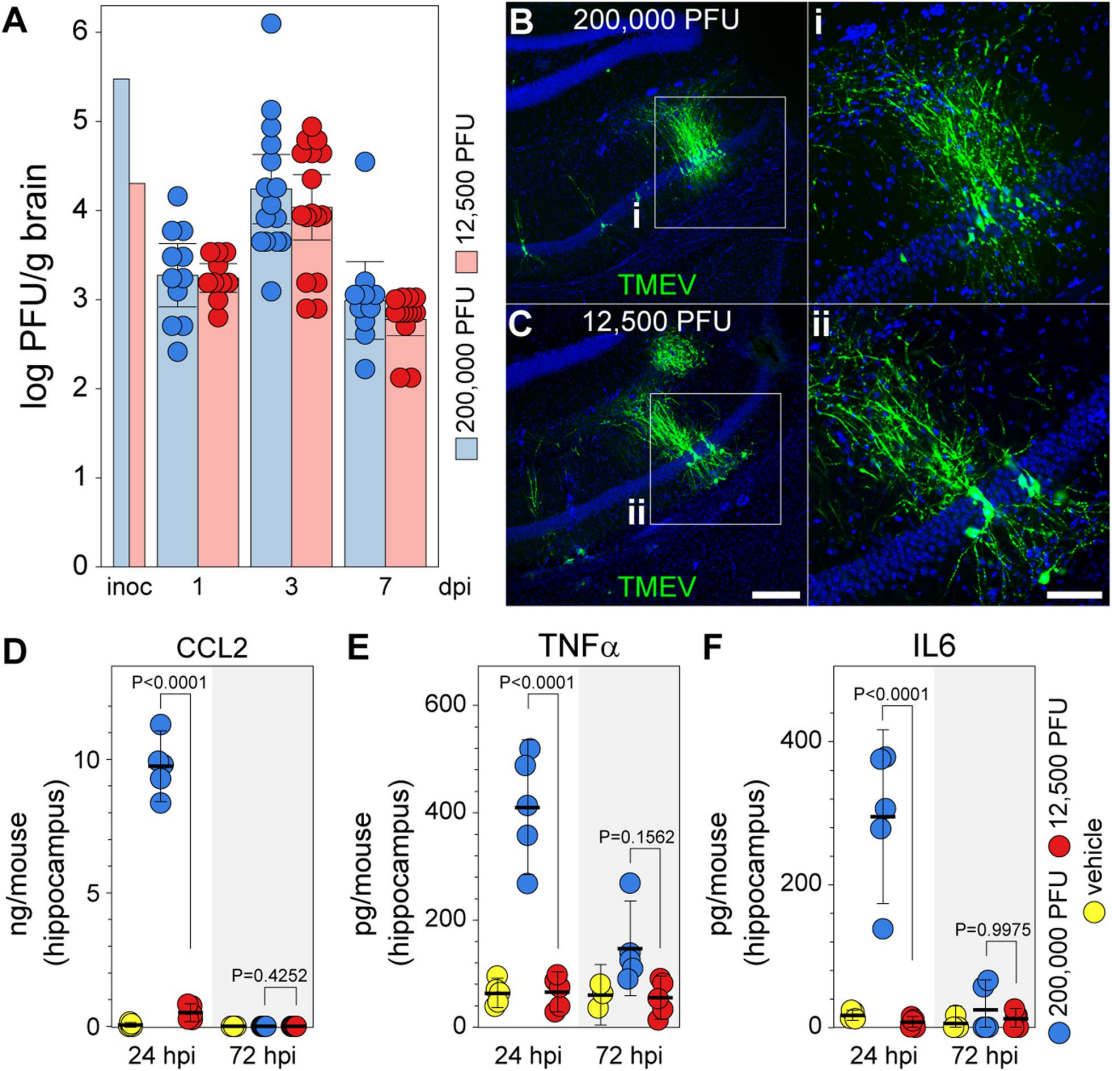
Viral load in the brain is not reduced in the low inoculum group, but inflammatory chemokine and cytokine production are reduced by more than 90% at 1 dpi. **A** Viral load was assessed at 1, 3, and 7 dpi in mice inoculated with 200,000 PFU or 12,500 PFU of TMEV. Despite a 16-fold reduction in the amount of virus delivered to the brain, the groups exhibit similar viral loads at each timepoint. *F*_(5,70)_ = 0.1534, *P* = 0.6964 between 200,000 PFU and 12,500 PFU by two-way ANOVA on ranks (inoculum x day); Shapiro–Wilk *P* = 0.0033; Tukey HSD pairwise analysis on ranks: 200,000 PFU vs 12,500 PFU @ 1 dpi: *P* = 0.6938; @ 3 dpi: *P* = 0.9900; @ 7 dpi: *P* = 0.9989; results from 5 separate experiments. **B**, **C** Representative images of TMEV immunostaining (green) in CA1 pyramidal neurons at 3 dpi in a mouse inoculated with 200,000 PFU (**B**) or 12,500 PFU (**C**) of virus; DAPI is shown in blue. Boxes indicate higher magnification insets “**i**” and “**ii**”. The amount of CCL2 (**D**), TNFα (**E**), and IL6 (**F**) in hippocampal homogenates was measured at 24 and 72 hpi. All of the factors were profoundly reduced at 24 hpi in the mice inoculated with 12,500 PFU of TMEV relative to mice inoculated with 200,000 PFU, with TNFα and IL6 indistinguishable from vehicle control mice. **D** CCL2, *F*_(2,12)_ = 318.3, *P* < 0.0001 by two-way ANOVA; Shapiro–Wilk *P* = 0.8826 (24 hpi); Tukey’s pairwise analysis: 24 hpi: 200,000 PFU vs 12,500 PFU: *P* < 0.0001; 200,000 PFU vs vehicle: *P* < 0.0001; 12,500 PFU vs vehicle: *P* = 0.5123; 72 hpi: 200,000 PFU vs 12,500 PFU: *P* = 0.4512; 200,000 PFU vs vehicle: *P* = 0.9999; 12,500 PFU vs vehicle: *P* = 0.5252; *d*_*s*_ @ 24 hpi = 11.8; results from 3 separate experiments. **E** TNFα, *F*_(2,28)_ = 15.92, *P* < 0.0001 by two-way ANOVA; Shapiro–Wilk *P* = 0.7674; Tukey’s pairwise analysis: 24 hpi: 200,000 PFU vs 12,500 PFU: *P* < 0.0001; 200,000 PFU vs vehicle: *P* < 0.0001; 12,500 PFU vs vehicle: *P* > 0.9999; 72 hpi: 200,000 PFU vs 12,500 PFU: *P* = 0.1562; 200,000 PFU vs vehicle: *P* = 0.3342; 12,500 PFU vs vehicle: *P* > 0.9999; *d*_*s*_ @ 24 hpi = 4.6; results from 3 separate experiments. **F** IL6, *F*_(2,28)_ = 28.975, *P* < 0.0001 by two-way ANOVA; Shapiro–Wilk *P* = 0.5052; Tukey’s pairwise analysis: 24 hpi: 200,000 PFU vs 12,500 PFU: *P* < 0.0001; 200,000 PFU vs vehicle: *P* < 0.0001; 12,500 PFU vs vehicle: *P* = 0.9993; 72 hpi: 200,000 PFU vs 12,500 PFU: *P* = 0.9975; 200,000 PFU vs vehicle: *P* = 0.9914; 12,500 PFU vs vehicle: *P* > 0.9999; *d*_*s*_ @ 24 hpi = 4.15; results from 3 separate experiments. Scale bar in **C** is 200 μm and refers to **B**; scale bar in **i** is 100 μm and refers to **i**

**Fig. 4:**
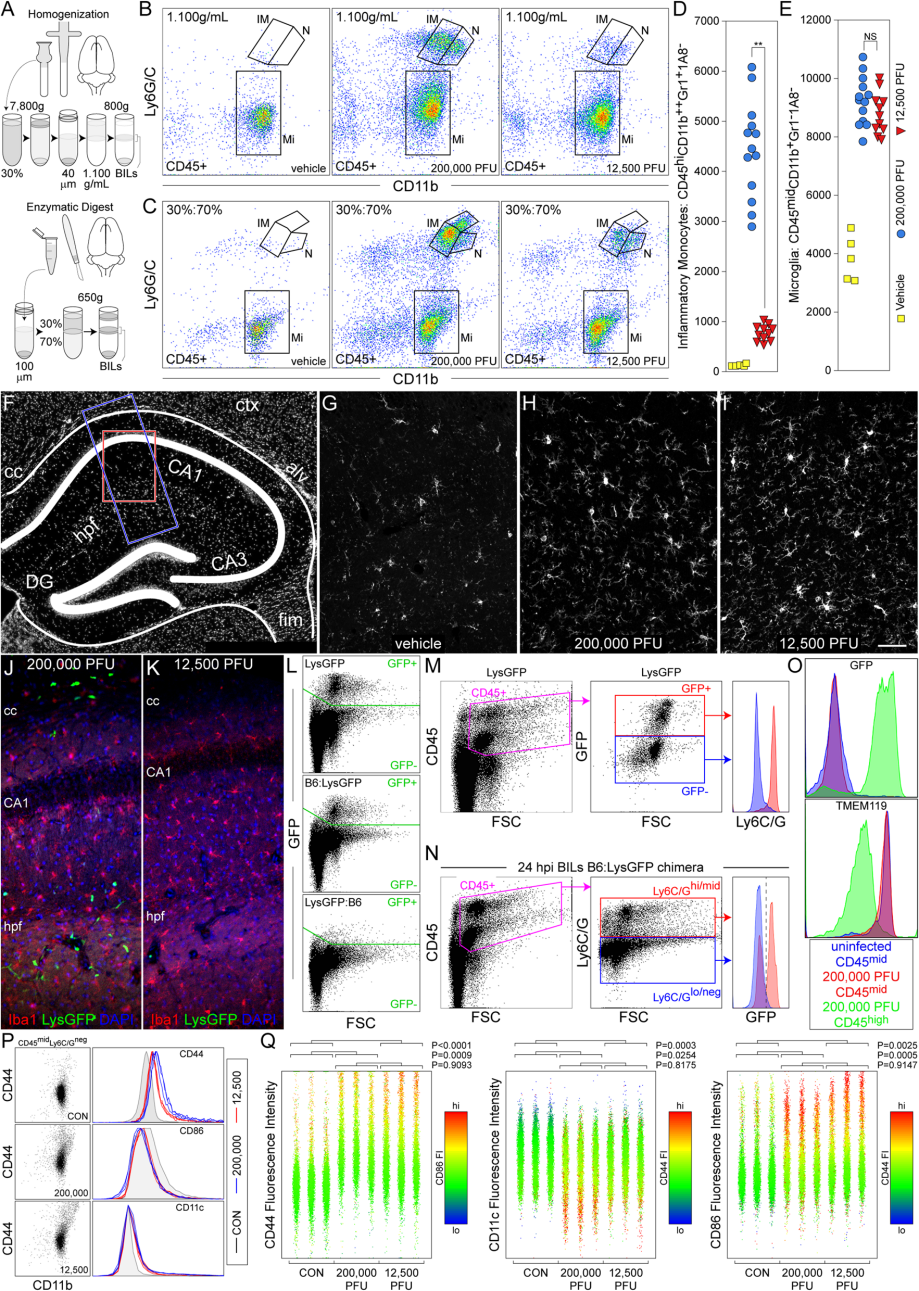
A 16-fold reduction in initial viral inoculum results in 80% attenuation of the brain-infiltrating inflammatory monocyte response but maintenance of microglial activation. Mice were inoculated with 200,000 PFU or 12,500 PFU of TMEV and processed for BILs isolation (**A**–**E**, **L–Q**) or immunostaining (**F**–**K**) at 24 hpi. (**A**) Schematic of the two different cell isolation protocols employed in the study. Cells shown in **B** and analyzed in **D**, **E** were prepared with homogenization and a 1.100 g/mL Percoll gradient; cells shown in **C** were prepared with enzymatic digestion and a 30%:70% gradient. **B** BILs isolated at 24 hpi from mice inoculated with 200,000 PFU of TMEV are enriched in CD45^hi^CD11b^++^Gr1^+^1A8^−^ inflammatory monocytes (IM) and CD45^hi^CD11b^++^Gr1^+^1A8^+^ neutrophils (N) as compared to vehicle-inoculated mice. Both populations are substantially reduced in mice inoculated with 12,500 PFU of TMEV. CD45^mid^CD11b^+^Gr1^−^1A8^−^ microglia (Mi) isolated by the gradient are increased in both 200,000 PFU and 12,500 PFU inoculations relative to vehicle control mice. (**C**) The same pattern is observed using the alternative cell isolation method. **D** Inflammatory monocytes: *F*_(2,26)_ = 121.5718, *P* < 0.0001 by one-way ANOVA; Shapiro–Wilk *P* < 0.0001; Dunn's method pairwise analysis: 200,000 PFU vs 12,500 PFU: *P* = 0.0019; 200,000 PFU vs vehicle: *P* < 0.0001; 12,500 PFU vs vehicle: *P* = 0.2616; *d*_*s*_ = 2.9; results from 4 separate experiments. **E** Microglia: *F*_(2,26)_ = 94.6556, *P* < 0.0001 by one-way ANOVA; Shapiro–Wilk *P* < 0.0001; Dunn's method pairwise analysis: 200,000 PFU vs 12,500 PFU: *P* = 0.8054; 200,000 PFU vs vehicle: *P* = 0.0009; 12,500 PFU vs vehicle: *P* = 0.0234; *d*_*s*_ = 2.6; results from 4 separate experiments. ** in panel (**D**) indicates *P* < 0.01; NS = not significant. **F** Schematic showing the location of immunostaining analysis in **G**–**I** (red box) and (**J**, **K**) (blue box). Iba-1^+^ activated microglia are shown in single channel (**G**–**I**) and in red with DAPI-labeled nuclei (blue) (**J**, **K**). Inoculation with 200,000 PFU of TMEV induced a large upregulation in Iba-1 expression in the stratum radiatum of CA1 (**H**) as compared to baseline levels in vehicle-inoculated mice (**G**). A similar increase in microglial activation is observed in mice inoculated with 12,500 PFU of TMEV (**I**). Analysis of LysGFP mice revealed strong Iba1^+^ microglia and GFP^+^ inflammatory monocytes in mice inoculated with 200,000 PFU TMEV (**J**) and equivalent Iba1^+^ microglia but absent GFP^+^ cells in mice inoculated with 12,500 PFU (**K**). Scale bar in **I** is 50 μm and refers to **G**–**I**. **L** Bone marrow chimerics were generated by reconstituting irradiated C57BL/6 recipient mice with bone marrow from LysGFP donor mice (B6:LysGFP) or LysGFP recipient mice with bone marrow from C57BL/6 donor mice (LysGFP:B6). B6:LysGFP mice exhibited GFP^+^ brain-infiltrating cell levels comparable to non-chimeric LysGFP mice, while LysGFP:B6 mice showed almost no GFP^+^ infiltrate. **M** Gating CD45^+^ cells in the cellular suspension from LysGFP mice indicated that GFP^neg^CD45^+^ cells (blue box and histogram) were Ly6C/G negative while GFP^+^CD45^+^ cells (red box and histogram) were Ly6C/G bright. **N** The specific association of Ly6C/G with inflammatory monocytes, not microglia, was confirmed by applying the same gates to the cellular suspension from B6 recipients receiving bone marrow from LysGFP donors. **O** CD45^mid^ cells are GFP^neg^ and TMEM119^+^ while CD45^hi^ cells are GFP^+^ and TMEM119^neg^. **P** CD45^mid^Ly6C/G^neg^ microglia exhibited increased levels of the CD44 and CD86 activation markers in mice inoculated with either 200,000 PFU or 12,500 PFU TMEV. CD11c levels are unchanged on this population. **Q** Two-dimensional expression analysis in CD45^mid^Ly6C/G^neg^ microglia: expression level of first label shown along y-axis; expression level of second label shown by color coding; each dot represents a single cell. CD44^hi^CD86^hi^ microglia are equivalently increased in mice inoculated with either 200,000 or 12,500 PFU TMEV; the CD44 brightest cells were CD11c low or negative in both groups. Data are from 3 mice per condition. *P* values were calculated using the Kolmogorov–Smirnov test comparing the cumulative frequency distributions

All figures were created in Photoshop and Illustrator. Statistics are reported following Curran-Everett guidelines [45]. Data were analyzed and graphed in JMP Pro 14.1 or GraphPad Prism 9.0 with α = 0.05 established a priori. All bar graphs show the mean and all error bars represent 95% confidence intervals (CI). Where relevant, each symbol represents one animal. Flow plots in Figs. 1 and 4 are representative of at least 5 mice in a minimum of two separate experiments. Histology in Fig. 2 is representative of at least 3 mice per group in two separate experiments. Immunostaining in Figs. 3 and 4 is representative of at least 3 mice in two separate experiments. For all quantitative analyses the number of mice are indicated in the statistics provided in the figure legends. Data were analyzed by one-way or two-way ANOVA as appropriate to the experimental design using a linear least-squares fit. Normality was assessed by Shapiro–Wilk test. Tukey’s multiple comparison was used for parametric data. Dunn's non-parametric pairwise comparison was used for all one-way ANOVA tests on data that were not from the normal distribution. For two-way ANOVA tests on non-parametric data, the data were stabilized using a Box–Cox power transformation [46] prior to application of the Tukey HSD (honestly significant difference) pairwise comparison test. For non-parametric data that included zeros, the values were first recast as the reciprocal log_10_ of the value plus one before the power transformation. For non-parametric data that could not be transformed, Dunnett’s least-squares mean analysis versus control was employed for pairwise comparisons. Comparisons of cumulative distribution frequencies used the Kolmogorov–Smirnov test. All analyses were subjected to post hoc power analysis on the noncentral *F* distribution using β = 0.2 and experiments were only reported if sufficient power was attained (π ≥ 0.8). Cohen's standardized effect size *d*_*s*_ is also reported for experiments to establish the magnitude of the statistical claim [47]. Line length comparisons were performed using a nested one-way ANOVA with multiple comparisons corrected using the two-stage linear step-up procedure of Benjamini, Krieger and Yekutieli to yield the false discovery rate (*q*-value) [48].

## Results

### The size of the inflammatory monocyte response to acute TMEV infection scales with the size of the inoculum

LysM:eGFP reporter mice were intracranially inoculated with 200,000 PFU, 50,000 PFU, 12,500 PFU, or 3125 PFU of TMEV in 10 μL (fourfold dilution series). Mice were killed at 24 hpi and BILs were prepared using our standard method [35]. Cells were immunolabeled for CD45 and analyzed by flow cytometry (Fig. 1). As we previously described [24], inoculation with 200,000 PFU TMEV induced robust infiltration of CD45^hi^ cells that were readily detectable by forward and side scatter profile (Fig. 1A, 1F). This inoculum was also marked by a large population of infiltrating inflammatory monocytes, defined by a GFP^mid^ profile (Additional file 1: Fig. S1), and by a smaller population of infiltrating neutrophils, defined by a GFP^hi^ profile (Fig. 1K, P, Q). Inoculation with fourfold less virus (50,000 PFU) still resulted in a clear population of CD45^hi^ infiltrating cells (Fig. 1B, G) and a sizable inflammatory monocyte population (Fig. 1L, P, Q). Inoculation with 16-fold less virus (12,500 PFU) also still induced infiltration, with a forward-side scatter profile that was very similar to 50,000 PFU (Fig. 1C) and clearly detectable CD45^hi^ (Fig. 1H) and GFP^+^ populations (Fig. 1M, P, Q. Finally, inoculation with 64-fold less virus (3,125 PFU) resulted in only a low level of CD45^hi^ infiltrating cells (Fig. 1D, I) and very few GFP^+^ cells (Fig. 1N, P, Q), though the amount was still higher than occurs in sham-inoculated mice (Fig. 1E, J, O–Q) [24]. We conclude that the relative size of the inflammatory monocyte response induced by TMEV inoculation scales with the amount of infectious virus in the inoculum but that even two logs less virus is sufficient to induce measurable inflammatory infiltration.

### The amount of hippocampal injury induced by acute TMEV infection scales with the size of the inoculum

As we have previously reported, inoculation of B6 mice with 200,000 PFU TMEV results in profound neural injury largely restricted to the CA1 layer of the dorsal hippocampus [24, 25] (Fig. 2A, E, I, O). At 7 dpi, this injury typically results in loss of more than 50% of CA1 pyramidal neurons in both hemispheres and clear evidence of ongoing tissue injury and infiltration between the hippocampal fissure and the white matter tract overlying the hippocampus. Even in areas of relative preservation, the CA1 layer is marked by a plethora of dead or dying cells in mice inoculated with 200,000 PFU TMEV (Fig. 2I). To quantify this neural injury, the percentage of CA1 exhibiting clear pyramidal neuron loss at low magnification at approximately level 285 in the Allen brain atlas (e.g., Fig. 2A) is converted to a number between 0 and 10 (100% injury to the layer in one hemisphere = 10) and the hemispheric values are summed. As shown in Fig. 2O, 10 out of 10 mice exhibited scores of 9 or greater at 7 dpi following inoculation with 200,000 PFU TMEV. Fourfold reduction in the viral inoculum resulted in a general reduction in CA1 injury (Fig. 2B, F, O) and 16-fold less virus resulted in clear preservation of CA1 neurons (Fig. 2C, G, J), with 8 out of 8 mice exhibiting total scores less than 5 and 3 of 8 mice exhibiting no CA1 cell loss (Fig. 2O). Of note, CA1 neurons were preserved in mice inoculated with 12,500 PFU TMEV despite evidence of infiltrating cells within the pyramidal layer (arrowheads, Fig. 2J) and above the hippocampal fissure (arrowheads, Fig. 2G) at 7 dpi (predominantly lymphocytes), reflective of a productive anti-viral response [49, 50]. Further reduction of the viral inoculum to 3125 PFU per mouse resulted in essentially no hippocampal injury (Fig. 2D, H, O). Finally, the general pattern of injury was maintained through 45 dpi, with the two highest inoculum groups showing distortion of the hippocampal architecture (Fig. 2K, L) and the two lowest inoculum groups exhibiting maintenance of the architecture (Fig. 2M, N). We conclude that the amount of injury to the CA1 pyramidal neuron layer induced by TMEV inoculation scales with the amount of infectious virus in the inoculum. We also conclude that inoculation with 12,500 PFU TMEV results in robust preservation of CA1 and the hippocampal architecture. Based on the clear disparity between inoculation with 200,000 PFU (“normal”) and 12,500 PFU (“low”) these two conditions were used for the remainder of the study.

### Cognitive performance is maintained in mice inoculated with less TMEV

We have previously reported complete loss of spatial learning ability in B6 mice inoculated with 200,000 PFU TMEV using the Morris water maze [23, 25, 31, 32]. This finding was replicated using a modified Barnes maze test (Fig. 2P, Q). In contrast to mice in the 200,000 PFU TMEV group, animals inoculated with 12,500 PFU exhibited the same improvement in latency-to-escape over a 4-day testing period as vehicle-inoculated mice (Fig. 2P). Moreover, the low inoculum group, but not the 200,000 PFU TMEV group, exhibited the same reduction in error rate observed in vehicle-inoculated mice (Fig. 2Q). These results indicate that cognitive deficits scale with the size of the initial viral inoculum and hence with the size of the inflammatory infiltrate. Notably, learning is maintained in the animals inoculated with 12,500 PFU despite the presence of a reduced but detectable inflammatory infiltrate, suggesting that the mechanisms leading to CA1 neuron loss and consequent learning impairment require a threshold number of inflammatory monocytes.

### Viral load after infection is equivalent between inoculation groups, but acute inflammatory cytokine and chemokine levels are markedly different

The most parsimonious explanation for the observations above is that introduction of less virus into the brain results in less viral replication and therefore less inflammation and less injury. However, we found that the amount of infectious virus recovered from the brain at 1, 3, and 7 dpi was statistically identical between mice inoculated with 200,000 PFU TMEV and mice inoculated with 12,500 PFU TMEV (Fig. 3A). Moreover, the distribution of virus in the hippocampus at 3 dpi, as assessed by immunostaining, was identical between the inocula (Fig. 3B, C). This finding indicates that differences in viral replication and viral burden, per se, cannot explain the preservation of hippocampal neurons observed in the low inoculum group. On the other hand, while mice inoculated with 200,000 PFU TMEV produced roughly 10 ng/mouse of the inflammatory monocyte chemoattractant CCL2 in the hippocampus at 24 hpi, mice inoculated with 12,500 PFU produced less than 1 ng/mouse and only showed a small and insignificant increase relative to uninfected mice. By 3 dpi the levels of CCL2 in the hippocampus were at uninfected levels in both groups (Fig. 3D). Likewise, mice inoculated with 200,000 PFU TMEV produced 400 pg/mouse TNFα and 300 pg/mouse IL6 in the hippocampus at 24 hpi, while mice inoculated with 12,500 PFU showed no increase in these inflammatory cytokines above vehicle-inoculated controls (Fig. 3E, F). By 3 dpi the levels of hippocampal TNFα and IL6 in the high inoculum group had returned almost to control values and the low inoculum group remained at control levels. Notably, the amount of IFNγ in the hippocampus at 3 dpi was ~ 3 ng/mouse in animals inoculated with either 200,000 PFU TMEV (2826 ± 804 pg/mouse) or 12,500 PFU TMEV (3258 ± 733 pg/mouse), relative to less than 100 pg/mouse in the vehicle control group (46 ± 28 pg/mouse) (*F*_(2,12)_ = 7.692, *P* = 0.0071 by one-way ANOVA; high vs low inoculum: *P* = 0.8788; high vs vehicle: *P* = 0.0221; low vs vehicle: *P* = 0.0092 by Tukey’s multiple comparison test). These findings indicate that the initial viral burden in the brain controls acute inflammatory responses associated with TNFα and IL6 production in the hippocampus and that this inflammatory program is largely resolved by 3 dpi. In contrast, the host ability to mount a robust anti-viral IFNγ response at 3 dpi is independent of the initial inoculum and is consistent with the equivalent viral load at this timepoint. These observations also support our previous findings regarding CCL2 in the recruitment of inflammatory monocytes to the hippocampus in TMEV-infected mice [22] and suggest that the intensity of the inflammatory monocyte infiltrate dictates the amount of TNFα or IL6 generated in the infected hippocampus at 24 hpi.

### Microglial activation is equivalent between inoculation groups

To quantify differences in neuroinflammatory responses between inocula, we employed flow cytometric analysis of cells prepared using two different methods. In the first, we homogenized the brain and isolated cells over sequential Percoll gradients using our standard methodology (Fig. 4A, B, D, E, L–Q). We have shown that this preparation yields infiltrating inflammatory monocytes and neutrophils as well as resident microglia [25, 35]. In the second preparation, we used a modified enzymatic digestion and gradient method that is reportedly enriched for microglia [36] (Fig. 4A, C). Both preparations revealed an increase in the number of CD45^+^CD11b^+^Ly6G/C^neg^ microglia in response to inoculation with 200,000 PFU and 12,500 PFU TMEV, relative to vehicle inoculation (Fig. 4B, C). In the same analyses, the number of CD45^+^CD11b^++^Ly6G/C^+^ inflammatory monocytes was increased in mice inoculated with 200,000 PFU TMEV but was severely attenuated in mice inoculated with 12,500 PFU, consistent with the findings in Fig. 1. Further quantitative analysis revealed a significant reduction in the number of CD45^hi^CD11b^++^Ly6C/G^+^1A8^−^ inflammatory monocytes in mice inoculated with 12,500 PFU TMEV relative to inoculation with 200,000 PFU TMEV (Fig. 4D) but no significant change in the number of CD45^mid^CD11b^+^Ly6C/G^−^1A8^−^ microglia between the groups (Fig. 4E). The equivalent microglial response was also revealed by increased Iba-1 immunoreactivity and the presence of hyper-ramified Iba-1^+^ cells in mice inoculated with either 200,000 PFU (Fig. 4H) or 12,500 PFU (Fig. 4I) relative to control mice (Fig. 4G). This finding was replicated in LysGFP reporter mice, with animals inoculated with 200,000 PFU showing microglial activation (increased Iba1^+^) and GFP^+^ infiltrate (Fig. 4J), while mice inoculated with 12,500 PFU showed equivalent microglia activation but no GFP^+^ cells (Fig. 4K).

**Fig. 5:**
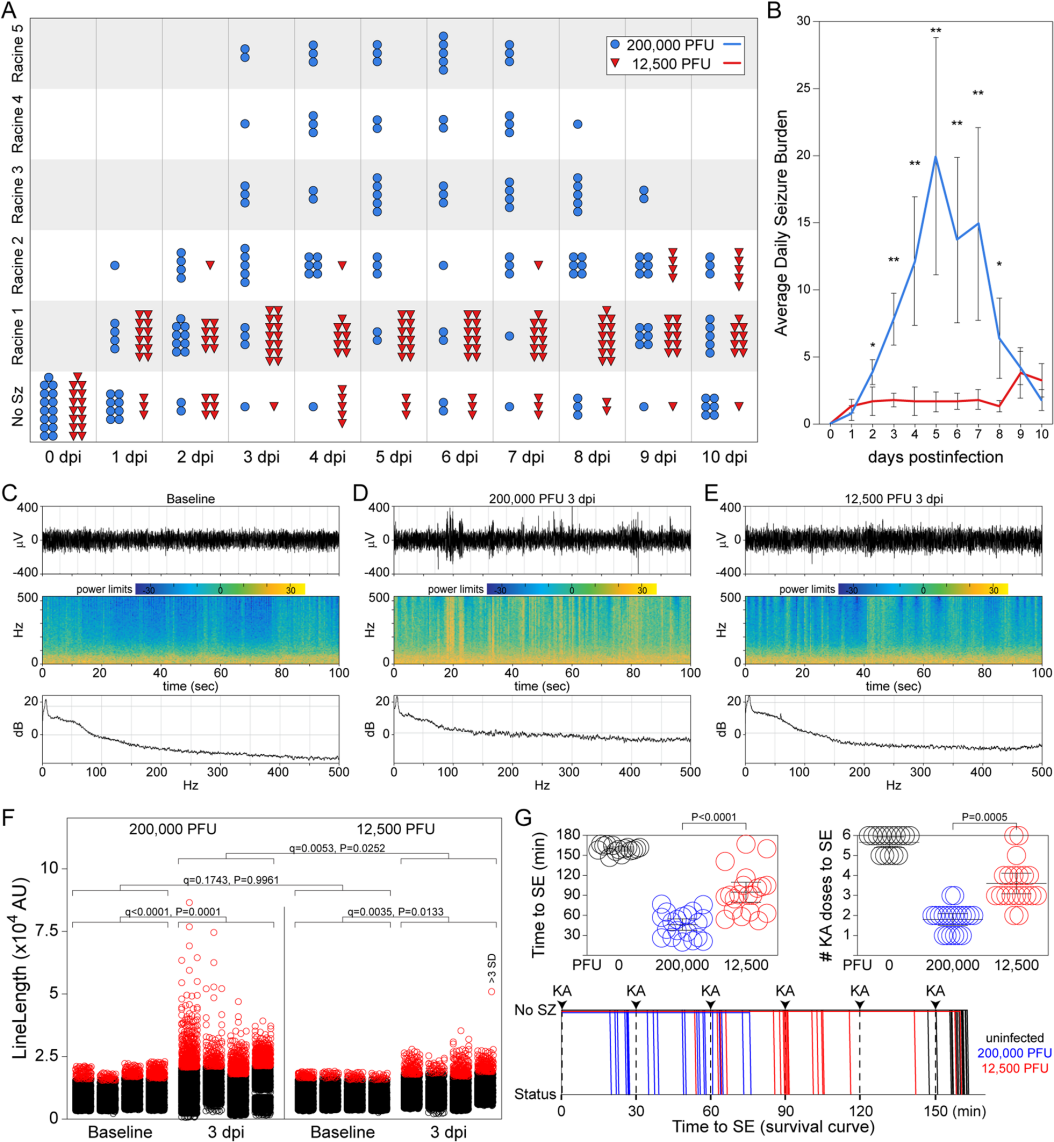
Seizure severity and frequency are highly attenuated in mice inoculated with 16-fold less TMEV. Seizure activity was assessed from 0 to 10 dpi using Racine scoring (**A**, **B**). **A** Racine seizure scoring (max score per daily epoch per mouse) indicates that mice inoculated with 12,500 PFU TMEV experienced only low-level seizure events (Racine 1) while the majority of mice inoculated with 200,000 PFU exhibited Racine scores of 2 or higher between 3 and 8 dpi. **B** The overall average daily seizure burden per group also indicates attenuation of behavioral seizure events in mice inoculated with 12,500 PFU TMEV. Average burden: *F*_(21,308)_ = 14.5642, *P* < 0.0001 by two-way ANOVA on ranks (inoculum x day); Shapiro–Wilk *P* < 0.0001; Dunnett’s least-squares means differences pairwise at each day: * = *P* < 0.01; ** = *P* < 0.001; *d*_*s*_ = 0.74. Blue line = 200,000 PFU; red line = 12,500 PFU. **C**–**E** Representative EEG traces, power spectra (power from 0 to 500 Hz), and spectrograms (short-time Fourier transform of the input) for a 100 s epoch at baseline (**C**) and at 3 dpi in an animal inoculated with 200,000 PFU (**D**) or 12,500 PFU (**E**). **F** EEG line lengths (1 s window) were calculated from 1.5 h epochs recorded at 1 day prior to inoculation (baseline) and again at 3 dpi in mice receiving 200,000 PFU or 12,500 PFU. Each symbol represents 1 s of EEG; values greater than 3 SD above the mean are shown in red. Data are from 4 mice in each group. *P* values were calculated using a nested one-way ANOVA with multiple comparisons corrected using the two-stage linear step-up procedure of Benjamini, Krieger and Yekutieli to yield the false discovery rate (*q*-value). **G** Seizure thresholds (time to status epilepticus (SE) induced by repeated KA injections) were assessed in control mice (black circles) and at 24 hpi in mice inoculated with 200,000 PFU (blue circles) or 12,500 PFU (red circles). Each symbol represents one animal; data are pooled from 4 experiments. Survival curve shows timing of doses and time to SE for each animal. Time to SE was analyzed by one-way ANOVA with Tukey’s pairwise comparison; number of KA doses was analyzed by Kruskal–Wallis one-way ANOVA with Dunn’s pairwise comparison

The specific identification of microglia by flow cytometry within the context of infiltrating inflammatory monocytes remains a contentious matter. We have previously characterized microglia as CD45^mid^CD11b^+^Ly6C/G^−^1A8^−^ and inflammatory monocytes as CD45^hi^CD11b^++^Ly6C/G^+^1A8^−^. In order to verify these parameters and facilitate flow cytometric analysis of microglial activation markers, we employed bone marrow chimeric mice in which irradiated B6 hosts were reconstituted with LysGFP bone marrow (B6:LysGFP) or irradiated LysGFP hosts were reconstituted with B6 bone marrow (LysGFP:B6) (Fig. 4L–N). We verified that the CD45^+^GFP^+^ infiltrating cells isolated from the brain at 24 hpi following inoculation with 200,000 PFU TMEV were clearly separable from the CD45^+^GFP^−^ microglia by relative expression of Ly6C/G (Fig. 4M). We also verified the converse, that CD45^+^Ly6C/G^hi/mid^ infiltrating cells were clearly separable form CD45^+^Ly6C/G^lo/neg^ microglia by the relative expression of GFP in B6 mice reconstituted with LysGFP bone marrow (Fig. 4N). Using these parameters, we further verified that all CD45^mid^ cells were GFP^−^ and Ly6C/G^lo/neg^ cells were TMEM119^+^ microglia while all GFP^+^Ly6C/G^hi^ cells were TMEM119^−^ (Fig. 5O). These findings validate the use of CD45^mid^Ly6C/G^lo/neg^ gating to separate microglia from infiltrating cells. Comparison of CD44 expression on CD45^mid^Ly6C/G^lo/neg^ microglia at 24 hpi revealed that this marker, along with CD86, but not CD11c, was increased to the same extent in animals inoculated with either 200,000 PFU or 12,500 PFU TMEV (Fig. 4P). Quantitative analysis confirmed that microglia upregulated CD44 and CD86 activation markers to the same extent in response to either inocula (Fig. 4Q), indicating that in contrast to the inflammatory monocyte response, local activation of microglia is either insensitive to the inoculum-scaling factors that drive CCL2 production and/or is directly responsive to the amount of replicating virus in the brain rather than the initial amount of virus introduced into the brain.

### Microglial activation is sufficient to induce low-level Racine seizures and alter seizure threshold, but inflammatory monocyte infiltration is necessary to drive high-level seizures

We have previously reported that mice inoculated with 200,000 PFU TMEV develop severe behavioral seizures within 3 days of infection [23]. This finding was replicated in the current study, with the majority of mice in this group experiencing at least a Racine level 2 seizure each day between 3 and 8 dpi and more than half of the mice experiencing Racine 3 or higher seizures between 4 and 7 dpi (Fig. 5A). Moreover, the aggregate average seizure burden per mouse in the 200,000 PFU group reached a peak of 20 at 5 dpi (Fig. 5B). In contrast, inoculation with 12,500 PFU TMEV resulted in clear attenuation of seizure intensity, as reflected by the development of only Racine level 1 seizures in the majority of mice between 2 and 10 dpi (Fig. 5A). No animal in this group was ever observed experiencing a seizure above Racine level 2. Likewise, the aggregate average seizure burden at 5 dpi in the low inoculum group did not exceed 3 (Fig. 5B). The presence of Racine 1/2 events suggested that the low inoculum condition still resulted in electrophysiological abnormalities though not to the same extent as the high inoculation conditions. Mice were implanted with screw electrodes and EEG data were collected at baseline (prior to inoculation) and at 3 dpi. Compared to baseline EEG (Fig. 5C), both 200,000 PFU (Fig. 5D) and 12,500 PFU (Fig. 5E) inoculations resulted in dysregulated EEG, including evidence of increased power across a broad spectrum of frequencies. Given the difficulties in identifying specific ictal events in mice infected with TMEV, we compared line length in 1-s windows through a ~ 90-min recording epoch at baseline and on 3 dpi (Fig. 5F). Both 200,000 PFU and 12,500 PFU inoculations led to an increase in line lengths greater than 3 standard deviations above the mean (*F*_(3,12)_ = 13.82, *P* = 0.0003 by one-way ANOVA across groups; 200,000 vs baseline: *q* < 0.0001, *P* = 0.0001; 12,500 vs baseline: *q* = 0.0035, *P* = 0.0133). Of note, mice inoculated with 12,500 PFU TMEV exhibited fewer EEG periods with line lengths in excess of 3SD compared to mice inoculated with 200,000 PFU (*q* = 0.0053, *P* = 0.0252). These results suggested that microglial activation in the context of strongly reduced monocyte infiltration in mice inoculated with 12,500 PFU TMEV was sufficient to induce electrophysiological dysregulation, but was not sufficient to induce high-level Racine events or increased periods of high-frequency activity in the EEG. This further suggested that microglial activation in the context of reduced monocytic infiltration would alter the threshold to seizures induced by kainic acid. To test this, mice were inoculated with 200,000 or 12,500 PFU TMEV or with vehicle and then stimulated with repeated intraperitoneal kainic acid until induction of status epilepticus (SE; defined as two independent episodes of Racine 4/5 seizures) (Fig. 5G). While control mice required 5 or 6 doses and took 160 ± 3 min to reach SE, mice inoculated with 200,000 PFU reached SE after 2 doses and 46 ± 9 min. Notably, mice inoculated with 12,500 PFU required more doses of KA (*H*_(3)_ = 42.66, *P* < 0.0001 by Kruskal–Wallis one-way ANOVA; 200,000 vs 12,500: *P* = 0.0005 by Dunn’s pairwise analysis) and took longer to reach SE (95 ± 15 min; *F*_(2,52)_ = 106.1, *P* < 0.0001 by one-way ANOVA across groups; 200,000 vs 12,500: *P* < 0.0001 by Tukey’s pairwise comparison). This indicates that the seizure threshold was significantly higher in mice with only microglial activation relative to mice with both microglial activation and inflammatory monocyte infiltration. However, given that the response in mice inoculated with 12,500 PFU TMEV was also different from control mice (KA doses: 12,500 vs vehicle: *P* = 0.0080 by Dunn’s pairwise analysis; time to SE: 12,500 vs vehicle: *P* < 0.0001 by Tukey’s pairwise comparison), it is clear that microglial activation was sufficient to disrupt electrophysiological homeostasis.

## Discussion

Infection of wild-type B6 mice with the Daniel's strain of TMEV is a unique model of ictogenesis and epilepsy associated with acute viral encephalitis [18, 20]. This model is marked by a clear dependence upon neuroinflammation as the pathophysiological driver of hippocampal injury and seizure development, with evidence from multiple labs supporting a role for infiltrating inflammatory monocytes, activated microglia, and inflammatory cytokine production in the initiation and propagation of seizures. Despite variability in inoculation parameters and nomenclature across investigators, the general consensus is that acute infection with TMEV drives CCR2-dependent CNS infiltration of inflammatory monocytes that secrete proinflammatory cytokines such as TNFα and IL6, leading to hippocampal circuit dysregulation and seizure induction. Likewise, there is consensus that microglia are robustly activated by TMEV infection and play an important role in shaping anti-viral immune responses. However, the interaction of inflammatory monocytes and microglia and the relative contribution of each cell type to both hippocampal injury and ictogenesis remains unclear.

Löscher and colleagues recently showed that genetic deletion of either CCR2 or CX3CR1 did not impact viral clearance and did not prevent the development of seizures, but did prevent hippocampal injury [26]. However, while CCR2 knockout mice developed seizures at the same frequency as wild-type mice, the seizures were of reduced severity, leading the investigators to suggest that inflammatory monocytes exacerbate seizures and hippocampal neuropathology. Our findings support this contention and further suggest that microglial activation in response to TMEV inoculation drives a specific type of behavioral seizure that may be separable from the more severe seizures induced by inflammatory monocytes. Given the absence of TNFα and IL6 induction in mice inoculated with 12,500 PFU TMEV, our findings suggest that these cytokines, produced by infiltrating inflammatory monocytes, may generate the intense Racine level 3–5 seizures that are most readily visualized in this model. In contrast, the low-level, subtle Racine level 1–2 seizures are generated irrespective of inflammatory monocyte infiltration and may be driven by distinct microglia-mediated mechanisms. At the present time it is unknown whether there is a difference in the anatomical or cellular locus of these different seizure phenotypes and we do not know if one type is more associated with the development of epilepsy in this model.

Löscher and colleagues also showed that pharmacological depletion of microglia with PLX5622 exacerbated hippocampal injury and increased seizure progression, concomitant with an increase in IL6 production [28]. While the authors indicate that the absolute number of presumptive inflammatory monocytes (CD45^hi^CD11b^+^ cells) was not different in infected PLX5622-treated mice vs infected controls, the data are difficult to assess based on the very high level of such cells detected in the brain in uninfected mice by these investigators. Nonetheless, their findings suggest that microglia may “put the brakes” on inflammatory monocyte-mediated inflammatory processes that contribute to ictogenesis and hippocampal damage. Our data indicate that microglial activation, as marked by hyper-ramification and increased Iba-1 immunoreactivity [51], occurs even in conditions in which inflammatory monocyte infiltration and concomitant high-level behavioral seizures and hippocampal injury are strongly attenuated. While Löscher has importantly pointed out that a population of microglia acquire levels of CD45 that render them indistinguishable from infiltrating monocytes, it is critical to note that our flow cytometric assessment of monocytes vs microglia uses Ly6G/C expression in CD45^+^ cells to segregate the populations [52, 53]. We find that inoculation with 12,500 PFU TMEV generates the same microglial response as inoculation with 200,000 PFU TMEV but results in more than 80% attenuation of inflammatory monocyte infiltration. Given that seizure burden and intensity were strongly suppressed in mice inoculated with 12,500 PFU TMEV, we conclude that inflammatory monocytes directly drive these events. It remains to be determined whether microglia produce factors that limit inflammatory monocyte cytokine production and/or suppress inflammatory or oxidative aspects of the hippocampal environment that facilitate monocyte-mediated effector functions.

An unexpected observation in this study was the similarity in viral load at 1, 3, and 7 dpi in mice inoculated with either 200,000 PFU or 12,500 PFU TMEV. This finding suggests that there may be only a limited number of infectable targets and that even 12,500 PFU is saturating for these sites. Indeed, Fujinami and colleagues recently showed that inoculation with 4,000, 400, or 40 PFU of TMEV still resulted in the same level of TMEV antigen expression in the CA2 region (CA1/CA3 border) of the hippocampus [54]. While they did not measure infectious virions, this finding supports the contention that only a small amount of introduced virus drives the consequent viral proliferation. As we have previously pointed out, mathematical modeling suggests that only 20–2000 cells are infected by 3 hpi [22], and the stereotypical drop (> 100-fold) in recoverable infectious virus measured at 1 dpi (relative to the inoculum) indicates that most virus (> 99%) does not persist after inoculation (Fig. 3). In fact, recovery of approximately 2000 PFU per gram tissue at 1 dpi means that there are only about 1000 infectious plaque-forming units across the entire brain. Our previous immunohistochemistry and in situ hybridization analyses suggest that only tens of cells are infected in the hippocampus at 2 dpi [32] and the Fujinami finding, though the timepoint was not specified, supports this contention [54].

What is notable about this outcome is that only a small number of cells therefore have the capacity to respond to cytosolic viral RNA sensing mediated by receptors such as IFIH (MDA5) and DHX58 (LGP2) [55, 56]. The role for these RIG-I-like pathogen sensors is highlighted by the fact that inoculation with UV-inactivated TMEV does not drive CCL2 production and does not induce infiltration of inflammatory monocytes [22], indicating that TLR-mediated recognition of non-infectious viral particles does not directly contribute to monocyte recruitment. However, what is not clear is whether such TLR-mediated signaling may be involved in microglial activation following inoculation. Given the robust activation of microglia induced by 12,500 PFU TMEV, it is possible that these cells sense extracellular or endocytosed virus without the need for productive infection. In contrast, since 12,500 PFU TMEV only induced a small amount of CCL2 and since we previously showed that neurons are the primary source of CCL2 during acute infection [22], it is possible that productive infection and introduction of viral RNA into the neuronal cytoplasm is required for the induction of an inflammatory monocyte response. However, because inoculation with 12,500 PFU TMEV led to the same amount of infectious viral load by 24 hpi, any effect of RIG-I-like receptor signaling on monocyte recruitment must be restricted to the most acute phase of the infection. Our previous observation that CCL2 is induced in the brain at levels in excess of 4000 pg/mouse by 3 hpi supports this model [22]. Thus, while currently speculative, the dichotomy between CCL2 production via RIG-I-like receptor-mediated pathways and microglial activation via TLR pathways may establish the set-point for consequent neuroinflammatory sequelae. If sufficient virus is introduced into the brain to drive productive infection of neurons within the first several hours, RIG-I-like receptor signaling drives CCL2 production, recruitment of inflammatory monocytes, and the creation of a TNFα- and IL6-rich environment that dysregulates hippocampal synapses and leads to severe seizures. In parallel, TLR-mediated activation of microglia drives a response that modulates inflammatory monocyte-mediated effector functions, resulting in the observation that the absence of microglia exacerbates hippocampal injury and seizure severity. On the other hand, if only enough virus is introduced to acutely drive TLR signaling that activates microglia but not neurons, the environment, while still inflammatory, per se, does not lead to inflammatory monocyte recruitment and does not progress to high-level seizures, though low-level behavioral seizures are still induced.

Welsh and colleagues recently characterized the ictogenic effect of two different variants of the Daniel’s strain of TMEV that exhibit different growth characteristics on baby hamster kidney cells [30]. One variant grows as large plaques while the other is characterized by small plaques. In notable parallel with our current study, these investigators found that the different variants induced substantially different pathogenic and ictogenic outcomes following intracranial inoculation. While the small variant robustly replicated in the brain, the large variant did not. Likewise, the small variant drove more microglial activation than the large variant, as revealed by Iba-1 immunolabeling, and the small variant induced the type of intense CA1 hippocampal injury we describe in this study while mice inoculated with the large variant showed no such injury. Moreover, mice infected with the small variant had a larger inflammatory monocyte response and, critically, developed more severe seizures, with nearly 90% of small variant infected mice exhibiting Racine 5 seizures but less than 10% of large variant hosts showing the same. While the Welsh study did not exactly replicate our findings (for example, both microglial activation and inflammatory monocyte responses were impacted), it is interesting that the mechanism underlying the size variant difference may relate to mutations in the TMEV Leader protein and L* protein that are involved in viral evasion of host innate responses. Specifically, the large variant has mutations at sites that are expected to compromise the ability of TMEV to inhibit type I interferon responses, resulting in host suppression of productive infection [57]. Thus, it is possible that the large variant in the Welsh study was similar to our lower inoculum-induced response, in which virions are introduced that may drive TLR signaling on microglia but which are deficient in replication efficacy necessary to drive cytosolic receptor signaling in neurons. Ultimately, it will be necessary to characterize the specific in situ cellular anti-viral signal transduction events elicited by acute TMEV inoculation in order to determine which pathways may be involved in ictogenesis. In particular, it will be valuable to determine if specific signaling events may be amenable to pharmacological manipulation that leads to a reduction in inflammatory monocyte infiltration without compromising the innate immune pathways necessary for viral control [22, 23, 31]. Limiting bystander pathology while maintaining immunological integrity may not only lead to neuroprotection in patients with viral encephalitis, but may also open up new avenues for thwarting the neuroinflammatory mechanisms of ictogenesis across the epilepsies [58].

## Conclusions

Based on our findings, we conclude that the relative size of the inflammatory monocyte response induced at 24 hpi by TMEV inoculation scales with the amount of infectious virus in the initial inoculum, despite the development of equivalent infectious viral load by 24 hpi. In contrast, the microglial response to TMEV inoculation does not scale with the amount of infectious virus in the inoculum. We also conclude that CA1 pyramidal neuron injury and disruption of spatial learning scale with the initial inoculum, suggesting that these events are primarily driven by inflammatory monocyte-mediated processes. Moreover, while TMEV inoculation conditions that drive strong inflammatory monocyte infiltration result in the development of robust behavioral seizures between 3 and 8 dpi, the low inoculum condition, associated predominantly with microglial activation, drives the development of a subtle behavioral seizure phenotype that arises by 1 dpi and persists through at least 10 dpi. The differential but interdependent roles of infiltrating inflammatory monocytes and activated microglia suggest a model for acute-onset refractory status epilepticus in patients in which neuroinflammatory events alter the threshold to seizures while peripheral inflammatory mediators drive the brain over this threshold and into ictogenesis. Such a model has implications for designing new therapeutic strategies to stop refractory status epilepticus.

## Supplementary Information

**Additional file 1: Fig. S1**. Gating strategy for brain-infiltrating leukocyte analyses. Singlets are further refined by GFP intensity or CD45-positivity and then sub-gated on Gr1 or 1A8 and CD11b. Cells that are GFP bright are Gr1-positive and 1A8-positive neutrophils, while cells that are GFP mid are Gr1-positive 1A8-negative inflammatory monocytes.

## Data Availability

The datasets used and/or analyzed during the current study are available from the corresponding author on reasonable request.

## Abbreviations

PFU: Plaque-forming units
ANOVA: Analysis of variance
TMEV: Theiler's murine encephalomyelitis virus
DAPI: 4′,6-Diamidino-2-phenylindole
BILs: Brain-infiltrating leukocytes
H&E: Hematoxylin and eosin
dpi: Days post-infection
hpi: Hours post-infection
CCL2: Chemokine (C–C motif) ligand 2
TNFα: Tumor necrosis factor alpha
IL6: Interleukin-6
RT: Room temperature
DMEM: Dulbecco’s modified Eagle medium
PBS: Phosphate buffered saline
HBSS: Hanks' balanced salt solution
FBS: Fetal bovine serum
HEPES: 4-(2-Hydroxyethyl)-1-piperazine ethanesulfonic acid
BSA: Bovine serum albumin
FSC: Forward scatter
SSC: Side scatter
