# Supplementary material for: Inflammatory monocytes and microglia play independent roles in inflammatory ictogenesis
Source: J Neuroinflammation. 2022 Jan 29;19:22. doi: 10.1186/s12974-022-02394-1 (PMC8800194; doi:10.1186/s12974-022-02394-1)
Supplement: Supplementary file 1 — Additional file 1: Fig. S1. Gating strategy for brain-infiltrating leukocyte analyses. Singlets are further refined by GFP intensity or CD45-positivity and then sub-gated on Gr1 or 1A8 and CD11b. Cells that are GFP bright are Gr1-positive and 1A8-positive neutrophils, while cells that are GFP mid are Gr1-positive 1A8-negative inflammatory monocytes. [file 12974_2022_2394_MOESM1_ESM.pdf]

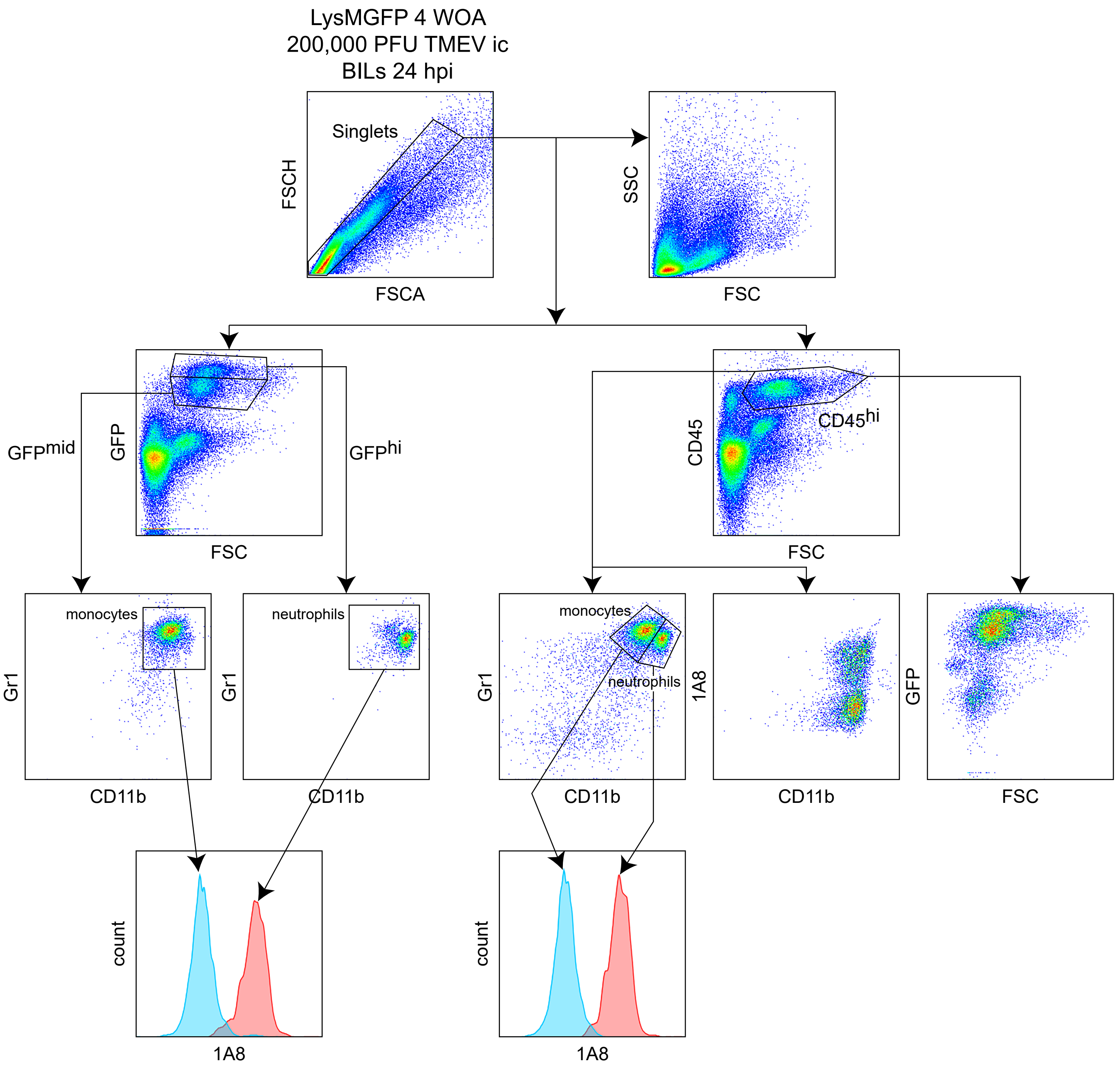

inflammatory monocytes = $CD45^{hi}GFP^{mid}Gr1^{++}1A8^{-}CD11b^{+}$

neutrophils = $CD45^{hi}GFP^{hi}Gr1^{+}1A8^{+}CD11b^{++}$

Supplemental Figure 1. Gating strategy for brain-infiltrating leukocyte analyses. Singlets are further refined by GFP intensity or CD45-positivity and then sub-gated on Gr1 or 1A8 and CD11b. Cells that are GFP bright are Gr1-positive and 1A8-positive neutrophils, while cells that are GFP mid are Gr1-positive 1A8-negative inflammatory monocytes.
